# Vitamin D concentration in maternal serum during pregnancy: Assessment in Hokkaido in adjunct study of the Japan Environment and Children’s Study (JECS)

**DOI:** 10.1371/journal.pone.0312516

**Published:** 2024-11-15

**Authors:** Ko Nakanishi, Mami Mutoh, Sachiko Itoh, Sumitaka Kobayashi, Takeshi Yamaguchi, Hiroyoshi Iwata, Naomi Tamura, Momoko Koishi, Machiko Kasai, Emi Kikuchi, Nanae Yasuura, Reiko Kishi, Yoshiaki Sato

**Affiliations:** 1 Faculty of Dental Medicine, Department of Biomaterials and Bioengineering, Hokkaido University, Sapporo, Hokkaido, Japan; 2 Faculty of Dental Medicine, Department of Orthodontics, Hokkaido University, Sapporo, Hokkaido, Japan; 3 Center for Environmental and Health Sciences, Hokkaido University, Sapporo, Hokkaido, Japan; 4 Division of Epidemiological Research for Chemical Disorders, National Institute of Occupational Safety and Health, Research Center for Chemical Information and Management, Kawasaki, Kanagawa, Japan; 5 Pediatrics, Hokkaido University Hospital, Sapporo, Hokkaido, Japan; University of Copenhagen: Kobenhavns Universitet, DENMARK

## Abstract

**Background:**

Vitamin D is an essential nutrient for maintaining blood calcium and phosphorus levels and controlling bone density. Deficiency in it leads to rickets, osteomalacia, osteoporosis, and various other diseases. Recently, it has gained attention for reportedly reducing the risk of COVID-19 severity. However, there are no reports evaluating pregnant women in the Hokkaido region of Japan. This study aims to elucidate the current status of vitamin D levels in pregnant women in the Hokkaido region.

**Methods:**

This study measured the serum concentrations of Vitamin D2 and D3 in 206 pregnant women participating in the Japan Environment and Children’s Study-Hokkaido sub-cohort at the Hokkaido Regional Center. It analyzed the relationship between these concentrations and the months, seasons, and sunshine hours.

**Results:**

The mean maternal age was 31.7 ± 4.7 years, and the mean prepregnancy BMI was 21.0 ± 2.5 kg/m^2^. Only two women have given birth at least once. Regarding sunscreen use, 65 participants (31.6%) responded “often” or “sometimes.” Five women used the supplement containing Vitamin D. The value of 25(OH)D2 was above 1 ng/ml in four of them. The average 25(OH)D3 level was 12.1 ng/ml, with a median of 11.0 ng/ml. Four participants (1.9%) had levels below 5 ng/ml. The highest median of 25(OH)D3 was in July, and the lowest was in April. The concentration of 25(OH)D3 was significantly higher in summer than in winter. A correlation was found between 25(OH)D3 and sunshine hours, with 25(OH)D3 concentrations gradually increasing as sunshine hours increase.

**Conclusion:**

It was found that almost all pregnant women in Hokkaido were deficient in vitamin D. It is necessary to implement measures to enhance vitamin D levels in pregnant women to safeguard the health of women and fetuses in Hokkaido.

## Introduction

Vitamin D is classified into two types: vitamin D2 (ergocalciferol) and vitamin D3 (cholecalciferol). Vitamin D2 is derived from plants such as mushrooms, while Vitamin D3 comes from animals such as fish and is also produced in the skin through exposure to sunlight [1]. They have different side chains but are metabolized similarly and have the same physiological effects. Both are metabolized to 25-hydroxyvitamin D in the liver, and this form is further metabolized to 1α,25-dihydroxyvitamin D, the active form, in the kidney. 1α,25-dihydroxyvitamin D binds to Vitamin D receptors in the nucleus of a target cell, inducing gene expression of Vitamin D-dependent proteins, thereby exerting its effects [2].

Vitamin D maintains blood calcium and phosphorus levels and controls bone density. A deficiency in it leads to rickets, osteomalacia, and osteoporosis [2]. Recently, vitamin D has been shown to have immunomodulatory and antiviral effects [3,4]. It has received further attention for its reported potential to reduce the risk of COVID-19 severity [5]. In addition, vitamin D is also known to be involved in common cancers, autoimmune diseases, infectious diseases, and cardiovascular diseases [6,7], and it is one of the important nutritional factors for leading a healthy life. Recently, due to concerns such as skin cancer prevention leading people to avoid sunlight, Vitamin D deficiency has become a concern [2,8]. Young women are particularly concerned about Vitamin D deficiency because they use sunscreen and sunshades to prevent skin tanning [9]. Pregnant women are similarly affected, as it is suggested they avoid going outside more because hormones during pregnancy can cause spots [10]. Maternal low vitamin D levels are associated with a higher risk of preeclampsia, gestational diabetes, preterm birth, cesarean section, low birth weight, asthma or recurrent wheezing, and impaired neurological development in children [11–14]. Yorifuji et al, [15] reported that craniotabes in normal newborns reflects a mild form of rickets *in utero* and occurs due to vitamin D deficiency in the pregnant woman, leading to vitamin D deficiency in the fetus. Vitamin D is an important nutrient during pregnancy. There are numerous reports about assessing vitamin D in pregnant women [16–18]. In Japan, the number of these reports is gradually increasing as the importance of vitamin D becomes clearer [19–22]. However, as far as we are aware, there is no report evaluating pregnant women in the Hokkaido region, Japan. It is crucial to assess the current status of vitamin D to safeguard the health of pregnant women and their fetuses.

**Fig 1:**
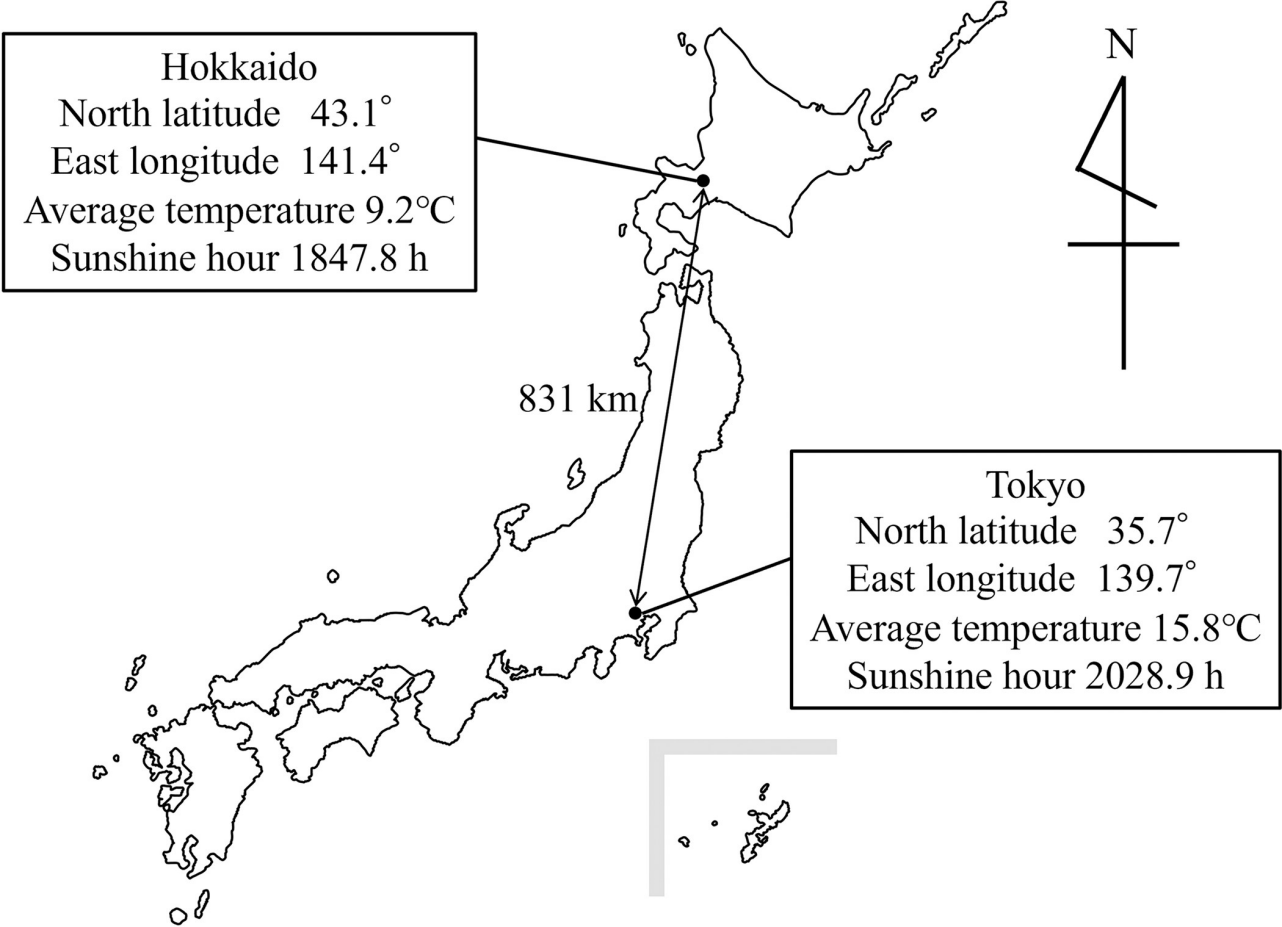
Location and climate of Hokkaido.

The Hokkaido region, situated at 43°N latitude, is Japan’s northernmost area and falls within the subarctic zone. The average temperature in Hokkaido is 9.2°C, the lowest in Japan (Fig 1). Moreover, Hokkaido is unique in Japan with its low typhoon risk, absence of a rainy season, and extended snowfall duration. It is known that vitamin D levels change with the seasons, generally being higher in the summer than in the winter [18], due to the body’s production of vitamin D being linked to sunlight exposure. In Hokkaido, snow covers the ground for about 4 months. In this season, it is risky for pregnant women to walk outdoors because they might slip or fall. Therefore, it is predicted that pregnant women have low vitamin D levels due to limited outdoor exposure, with significant differences in vitamin D levels between summer and winter.

This study aims to analyze vitamin D levels in pregnant women in the Hokkaido region and assess the impact of the climate in Hokkaido region. We measured the vitamin D levels in 206 pregnant women from the Hokkaido region and analyzed the differences across seasons.

## Material and methods

### 1. Study participants

**Fig 2:**
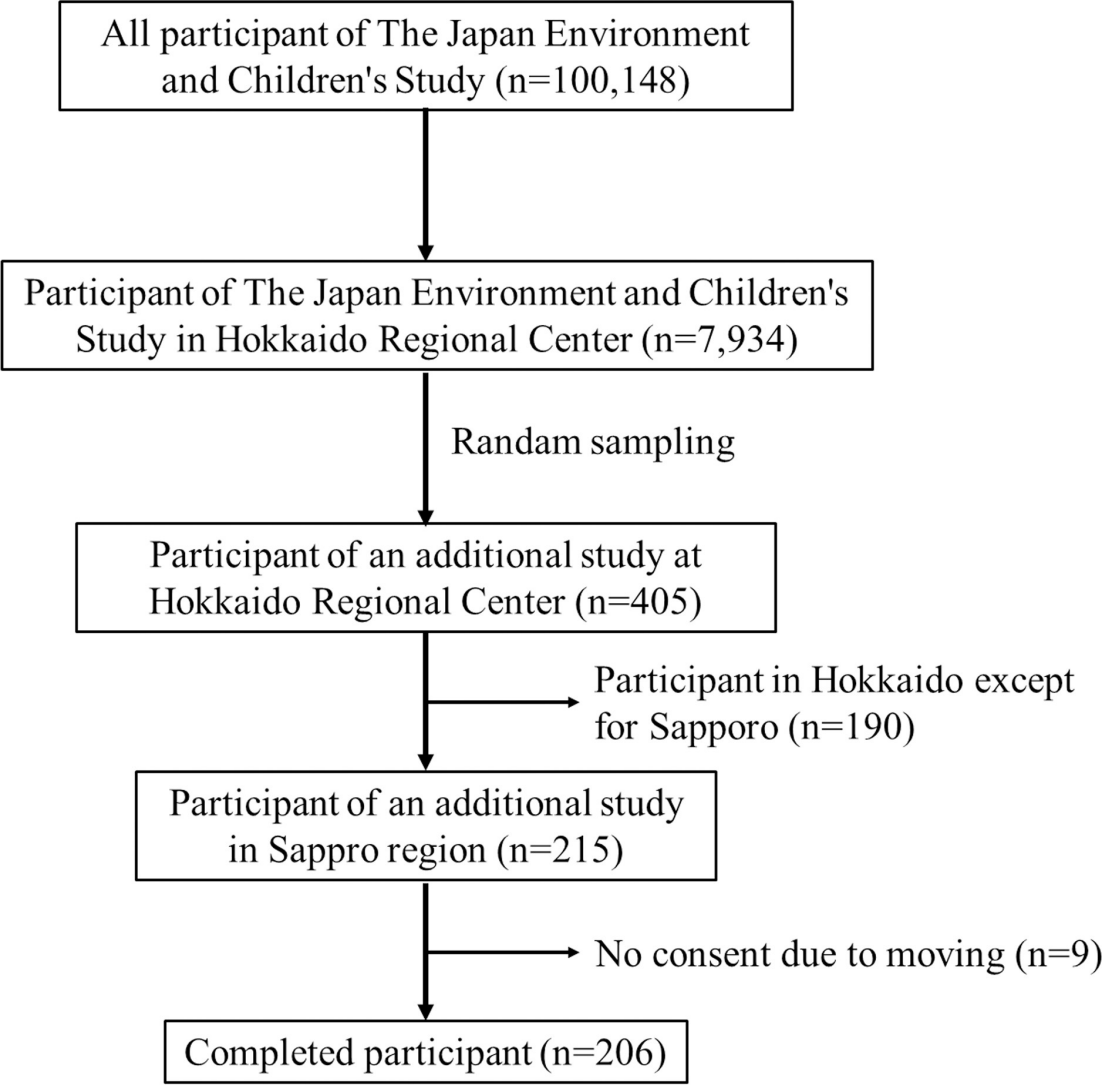
Study participants flow chart.

The Japan Environment and Children’s Study (JECS) is an ongoing prospective birth cohort study involving approximately 100,000 parents and their children across 15 regions in Japan. The JECS recruitment began in January 2011 and lasted until March 2014, spanning three years. Hence, the JECS is comprised of 15 regional subcohorts. At the Hokkaido Regional Center of the JECS, approximately 8,000 participants consist in the JECS-Hokkaido sub-cohort, which is part of the JECS cohort. Of the roughly 8,000 participants, 215 randomly selected individuals from the Sapporo region are under continuous investigation in an Additional Study. This study involved 206 participants (Fig 2). This study was conducted as an Additional Study at Hokkaido Regional Center in the JECS funded by the Ministry of the Environment, Japan. This study was approved by the Ministry of the Environment, Japan, the National Institute for Environmental Studies, and the ethics committee of the Center for Environmental and Health Sciences at Hokkaido University. Study participants were briefed on the study’s purpose and method, the voluntary nature of research cooperation, the freedom to withdraw, the benefits and disadvantages of participation, measures to protect personal information, and the handling and disposal of data. They were also informed about the methods of presenting research results and agreed to participate. The JECS received written consent from all participants. All studies were conducted in compliance with Declaration of Helsinki.

In this study, serum was collected by doctor, nurse or clinical laboratory technician from 206 mothers who participated in a detailed JECS survey at the Hokkaido Regional Center during the second or third trimester of pregnancy.

### 2. Measurement of Vitamin D

Serum samples were stored at −30°C at Hokkaido University until Vitamin D measurement. Serum concentrations of 25-hydroxyvitamin D2 (25(OH)D2), a metabolite of Vitamin D2, and 25-hydroxyvitamin D3 (25(OH)D3), a metabolite of Vitamin D3, were measured using liquid chromatography tandem mass spectrometry (LC-MS/MS) at LSI Medience (Tokyo, Japan). The obtained value of 25(OH)D3 was classified as normal for ≥30, shortage for 20 to <30, and deficient for <20 according to the method by Holick et al [23]. Deficiencies were also classified as mild for 10 to <20, moderate for 5 to <10, and severe for <5 according to the Gani and How method [24]. The obtained value of 25(OH)D3 was analyzed based on the month of sampling, the season of sampling, and the number of sunshine hours. In Sogawa’s study, spring is defined as March to May, summer as June to August, fall as September to November, and winter as December to February [19]. The sunshine hours over the 20 days prior to serum sampling were calculated based on data from the Japan Meteorological Agency [25].

### 3. Participant data

We collected the following mother data: Maternal age (<25, 25–30, 31–35, ≥36 years), parity (0, ≥1), and pre-pregnancy BMI (<18.5, 18.5–25.0, >25.0) were collected from medical records and questionnaires. Maternal body mass index (BMI) was calculated using the formula: body weight(kg)/(height(m))^2^. Annual household income (<2, 2 to <4, 4 to <6, 6 to <8, 8 to <10, and ≥10 million Japanese yen (JPY)), smoking habit (never smoked, quit smoking before pregnancy, quit smoking after pregnancy, and smoked during pregnancy), alcohol consumption (never drank, quit drinking before pregnancy, quit drinking after pregnancy, and drank during pregnancy), education (junior high school, high school, technical school, vocational school, junior college, university, and graduate school), use of vitamin D supplements (never, once or twice a week, third of fourth a week, more than fifth a week, everyday, and used supplements but unclear whether it contains vitamin D), use of sunscreen (never, rarely, sometimes, and often), work for agriculture, forestry and fisheries (yes and no) were obtained from self-administered questionnaires, which were written during the pregnancy.

### 4. Statistically analysis

When analyzing seasonal differences in 25(OH)D3, we assessed them using statistical methods based on their distribution. If the distribution is Gaussian, we use analysis of variance (ANOVA) to compare seasonal differences in 25(OH)D3. When detecting statistical significance in the ANOVA test with a P-value less than 0.5, we conducted a Student’s t-test to determine which seasons differed statistically. If the distribution was non-Gaussian, we used Kruskal–Wallis and Mann–Whitney U tests. We planned to conduct both statistical tests if we had difficulty evaluating the 25(OH)D3 distribution precisely. Further analysis used univariable linear regression to evaluate the relationship between 25(OH)D3 levels and hours of sunshine. All our analyses were performed using JMP Pro version 16.1.0 (SAS Institute Inc., Cary, NC, USA).

## Result

### 1. Participant characteristics

Table 1 shows the characteristics of mothers. The average maternal age was 31.7 ± 4.7 years, and 2 mothers (1.0%) had at least one previous birth. The average BMI prior to pregnancy was 21.0±2.5 kg/m^2^. About 60% of the participants had a household income between 2 and 6 million Japanese yen. Of the 121 mothers, 58.7% have never smoked, 56 (27.2%) quitted smoking before pregnancy, 17 (8.3%) quitted smoking after pregnancy, and 9 (4.4%) smoked during pregnancy. 128 mothers (62.1%) quitted drinking alcohol after pregnancy. The final education of participants was primarily vocational school (28.2%), followed by university (24.3%), high school (21.8%), junior college (16.0%), technical school (3.4%), junior high school (2.9%), and graduate school (2.4%). Regarding sunscreen use, 65 participants (31.6%) responded “often” or “sometimes.” Fifty-seven participants (27.7%) had never used sunscreen. Five mothers (2.5%) used the supplement containing Vitamin D. 1 mother (0.5%) work in agriculture, forestry, or fisheries.

**Table 1:** Characteristic of participants.

Characteristic		All	
		n	%
Maternal age (years), mean(SD)		31.7 (4.7)	
	<25	13	6.3
	25–30	73	35.4
	31–35	71	34.5
	≥36	46	22.3
	unkown	3	1.5
Parity			
	0	204	99.0
	≥1	2	1.0
BMI before pregnancy, mean (SD)		21.0 (2.5)	
	<18.5	27	13.1
	18.5–25.0	163	79.1
	>25.0	10	4.9
	unkown	6	2.9
Household income (Million Japanese yen)			
	<2	8	3.9
	2 to <4	65	31.6
	4 to <6	60	29.1
	6 to <8	38	18.4
	8 to <10	16	7.8
	≥10	11	5.3
	unkown	8	3.9
Smoking habit			
	never smoked	121	58.7
	quit smoking before pregnancy	56	27.2
	quit smoking after pregnancy	17	8.3
	smoked during pregnancy	9	4.4
	unkown	3	1.5
Alcohol consumption			
	never drank	46	22.3
	quit drinking before pregnancy	26	12.6
	quit drinking after pregnancy	128	62.1
	drank during pregnancy	4	1.9
	unkown	2	1.0
Education			
	junior high school	6	2.9
	high school	45	21.8
	technical school	7	3.4
	vocational school	58	28.2
	junior college	33	16.0
	university	50	24.3
	Graduate School	5	2.4
	unkown	2	1.0
Use of supplements containing vitamin D			
	never	186	90.3
	once or twice a week	3	1.5
	third or fourth a week	0	0.0
	more than fifth a week	0	0.0
	everyday	2	1.0
	Used supplements but unclear whether it contains vitaminD	13	6.3
	unkown (undescribed)	2	1.0
use of sunscreen			
	never	57	27.7
	rarely	16	7.8
	sometimes	65	31.6
	often	65	31.6
	unkown	3	1.5
Work for Agriculture, forestry or fisheries			
	Yes	1	0.5
	No	204	99.0
	unkown	1	0.5

### 2. Vitamin D status

The value of 25(OH)D2 above 1 ng/ml was found in 4 women, with a maximum of 1.6 ng/ml.

The average 25(OH)D3 level was 12.1 ng/ml, with a median of 11.0 ng/ml. The number of participants with 25(OH)D3 concentrations was 4 (1.9%) for 30 ng/ml or more, 11 (5.3%) for 20 ng/ml or more and less than 30 ng/ml, and 191 (92.7%) for below 20 ng/ml, as shown in Table 2. Additionally, 4 participants (1.9%) had levels below 5 ng/ml.

**Table 2:** Classification of vitamin D deficiency and distribution.

Vitamin D3 levels (ng/ml)			mother	
			number	%
Normal		≥30	4	1.9
Shortage		20 to <30	11	5.3
Deficiency	Mild	10 to <20	115	55.8
	Moderate	5 to <10	72	35.0
	Severe	<5	4	1.9

**Fig 3:**
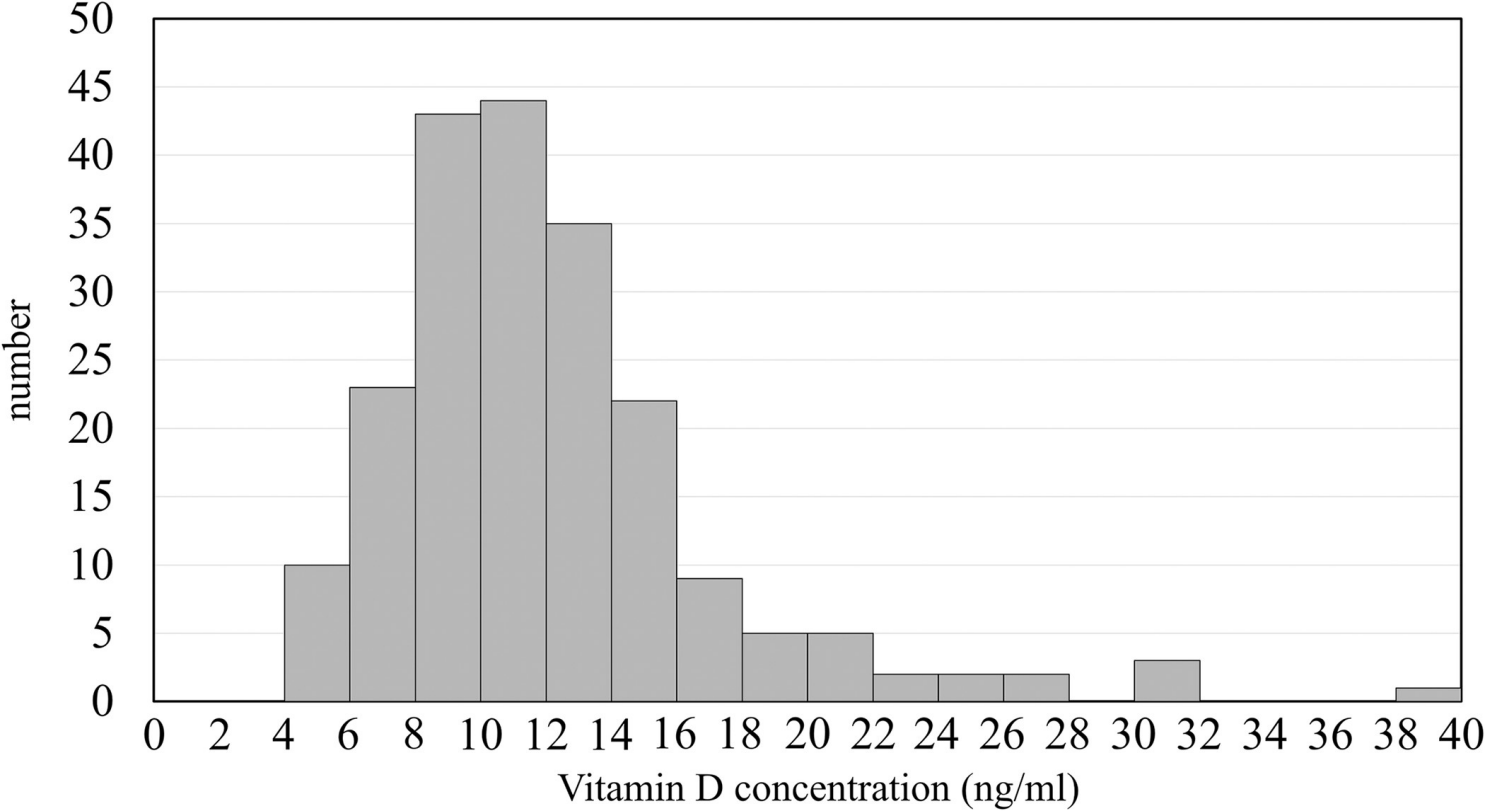
Vitamin D levels in pregnant women.

**Fig 4:**
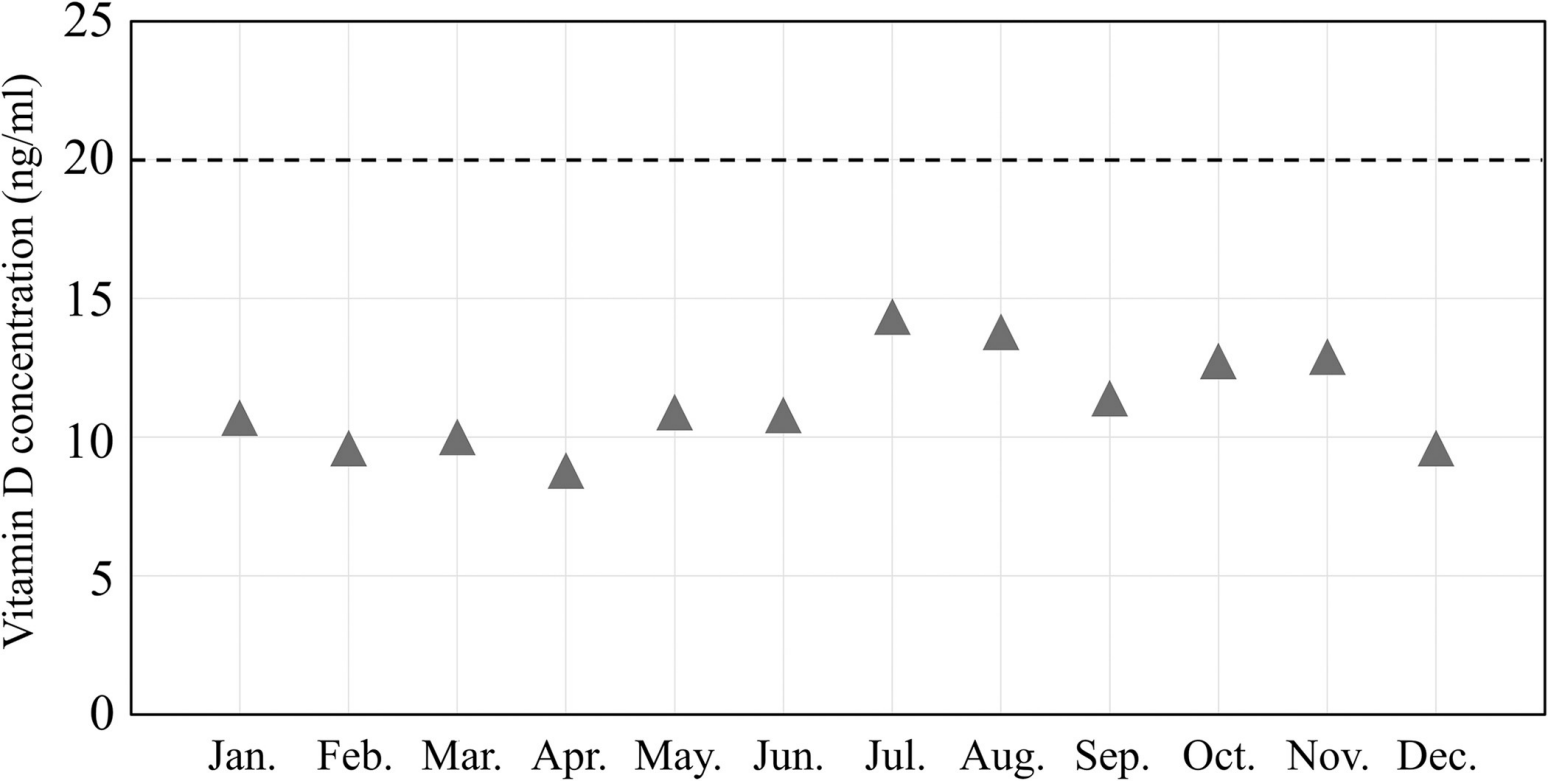
Monthy variations of vitamin D levels in pregnant women.

**Fig 5:**
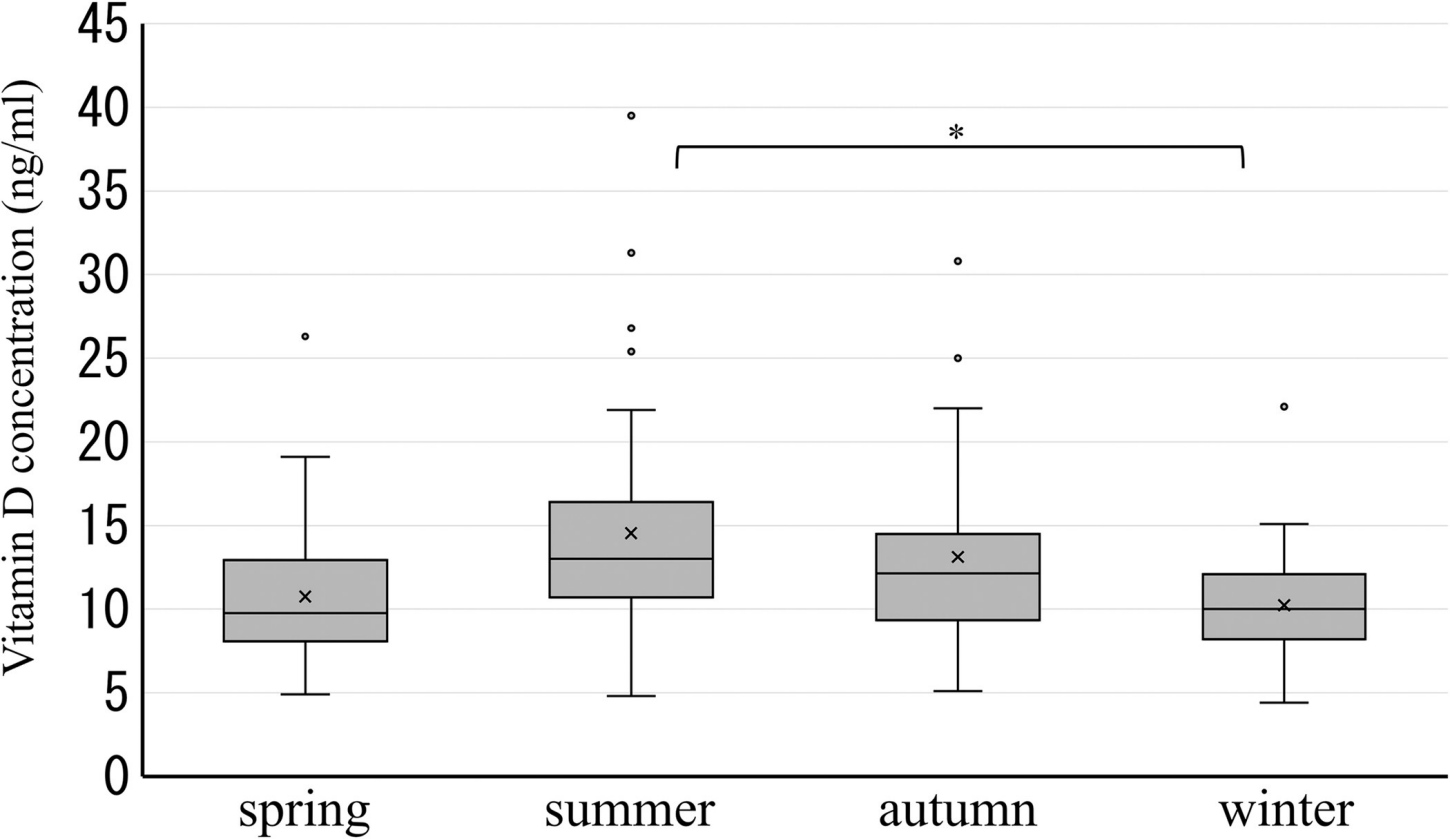
Seasonal vitamin D levels in pregnant women.

Fig 3 shows the distribution of Vitamin D levels among pregnant women. The number of participants with Vitamin D3 concentrations between 10 ng/ml and <12, 12, and <14 ng/ml, and 14 and <16 ng/ml were 43, 44, and 35, respectively, higher than in other ranges. The highest median of 25(OH)D3 was 14.4 ng/ml in July, and the lowest was 8.8 ng/ml in April (Fig 4). In season, the median level of 25(OH)D3 was highest in summer at 13.0 ng/ml and lowest in spring at 9.8 ng/ml. Seasonal variations in the concentrations of 25(OH)D3 were significantly different, as indicated by the ANOVA test (P-value < 0.0001). Additionally, the concentration of 25(OH)D3 in summer was significantly higher than in winter, with a P-value of <0.001 (Fig 5, Table 3). The Kruskal–Wallis and Mann–Whitney U tests also identify similar statistical significance in sensitivity analyses.

**Table 3:** Seasonal 25(OH)D3 value.

Season	Number	Average	medial
Spring	74	10.8±3.7	9.8
Summer	63	14.5±6.3	13.0
Autumn	26	13.1±5.8	12.2
Winter	43	10.2±3.3	10

**Fig 6:**
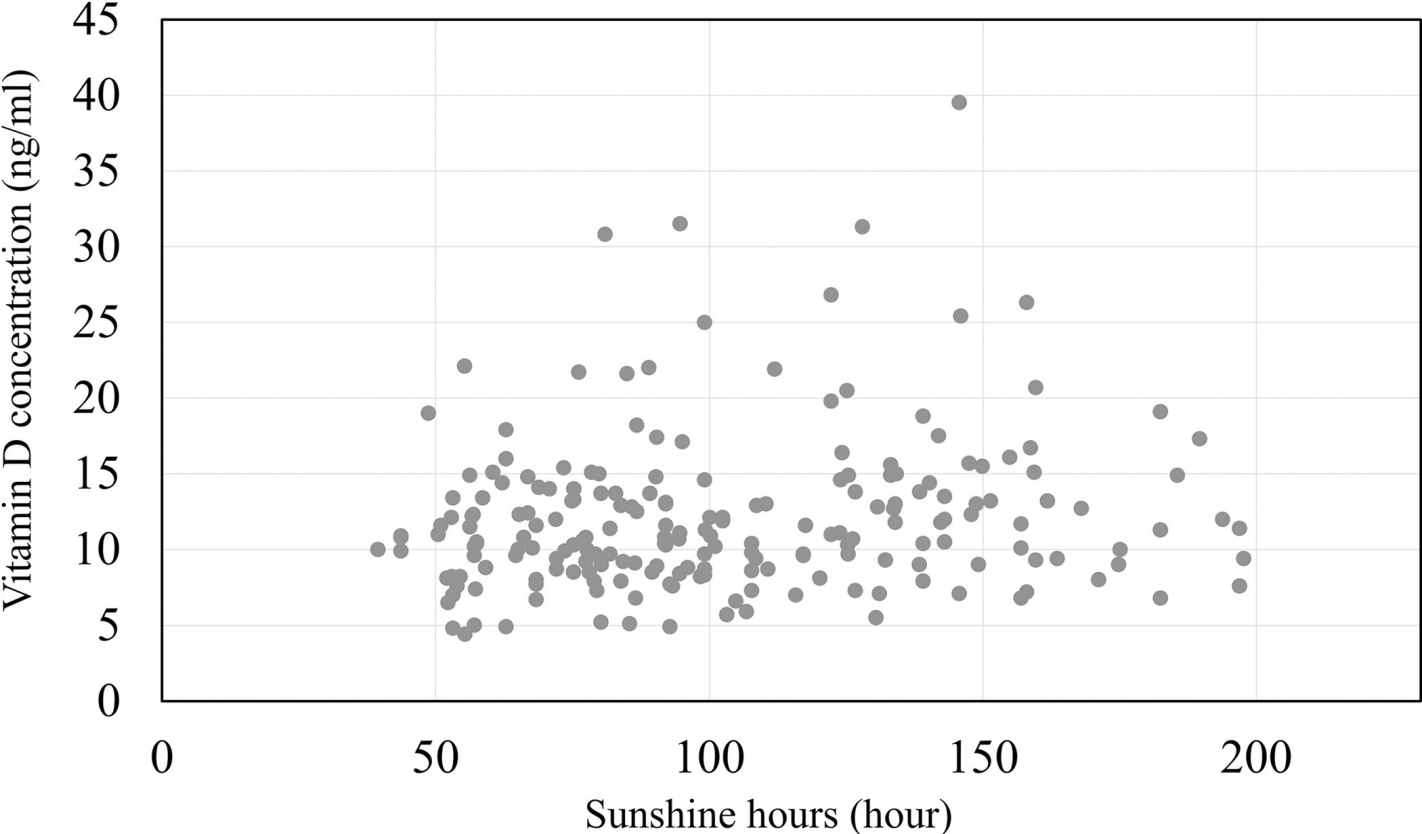
Correlation between vitamin D levels in pregnant women and sunshine hour.

The linear regression showed that the concentration of 25(OH)D3 was positively correlated with sunshine duration (β = 0.020; 95% CI, 0.0016 to 0.038) (Fig 6).

## Discussion

Except for four, all mothers had 25(OH)D2 levels below 1 ng/ml, which was under the detection limit. Machida et al. reported an average 25(OH)D2 level of 0.3 ng/ml in a study of 96 participants undergoing health examinations in Gunma, Japan from 2017 to 2018 [26]. Takatani et al. reported that the average 25(OH)D2 level among women 22 to 35 weeks pregnant in Chiba, Japan, was 0.2 ng/ml [21]. Their results are similar to those of this study, showing that the 25(OH)D2 levels in nearly all participants were below 1 ng/ml, indicating a very low intake of Vitamin D2. Vitamin D2 is obtained solely from plants, particularly mushrooms. Pregnant women are encouraged to eat mushrooms because they are rich in folic acid. Folic acid is very important for pregnant women because it aids in rapid cell division and growth [27]. However, the recommended intake of folic acid is too much to eat. Many mothers meet the recommended intake through supplements. In this study, 83 mothers took folic acid supplements. Therefore, it was assumed that the amount of eating mushrooms was reduced without needing to eat it for folic acid and the 25(OH)D2 level became quite low.

Many participants had low 25(OH)D3 levels, with an average of 12.1 ng/ml and a median of 11.0 ng/ml, but none were below the detection limit. In this study, the evaluation of Vitamin D levels was based solely on 25(OH)D3, as 98% of participants had undetectable 25(OH)D2 levels. It was reported that the mean 25(OH)D level of pregnant women was 11.4 ng/ml in Tokyo [22], 15.3 ng/ml in Chiba [21], 15.1 ng/ml in Kyoto, 16.1 ng/ml in Toyama, and 14.4 ng/ml in Tottori [20]. The 25(OH)D levels in pregnant women from Hokkaido were lower compared to other regions, except Tokyo. The difference in latitude is regarded as one of the reasons. The latitude of Hokkaido (43°N) is higher than that of other areas (Tokyo: 35.7°N, Chiba: 35.6°N, Kyoto: 35.0°N, Toyama: 36.7°N, Tottori: 35.5°N) in same Japan. Zamfiova et al. investigated the vitamin D levels of medical students in Florida (27°N) and Pennsylvania (42°N). They reported that the average vitamin D level was lower in Pennsylvania than in Florida, and latitude was found to be a statistically significant risk factor for vitamin D deficiency [28]. The reduction in vitamin D levels at higher latitudes is due to the increased oblique angle of the sun’s rays. The high oblique angle caused UVB photons, which produce Vitamin D when they irradiate the skin, to be efficiently absorbed by the ozone layer because the more oblique angle causes the UVB photons to pass through the ozone layer for a greater distance. It is known that the amount of UVB photons passing through the ozone layer decreases significantly from November to February at latitudes of 37 degrees north or higher [29]. Van der Mei et al. concluded that while latitude was related to vitamin D levels, behavioral factors were also important [30]. The lower vitamin D levels in mothers from Tokyo compared to those in Hokkaido might be attributed to behavioral factors. Summer temperatures in Tokyo, Chiba, Kyoto, Toyama, and Tottori are higher than those in Hokkaido. In these cities, the risk of heat stress is higher than in Hokkaido. Pregnant women are among the groups most vulnerable to heat stress [31]. Tokyo, the capital of Japan, likely has a higher interest in medical care among its residents compared to other regions. Pregnant women in Tokyo may have avoided outdoor activities to prevent heat stress, potentially resulting in lower vitamin D levels during the summer. However, because Nakajima et al. [22] did not report seasonal variations in Vitamin D concentrations, these details cannot be disclosed at this time. Further research is needed to identify factors in the future.

From a monthly perspective, July recorded the highest median 25(OH)D3 level at 14.4 ng/ml, followed by August with 13.8 ng/ml. On the contrary, April had the lowest median 25(OH)D3 level at 8.8 ng/ml, followed by February and November with levels of 9.6 ng/ml. In Sweden’s study [17], the median levels of 25(OH)D3 are highest in August, followed by September. The median levels of 25(OH)D3 are lowest in March, followed by April. These results are similar to our results. Vitamin D levels are generally higher in summer and autumn than in spring and winter. In this study, the median 25(OH)D3 level in summer was significantly higher than in spring and winter, and slightly higher than in autumn. It indicated that pregnant women and nonpregnant individuals show no difference in terms of increasing their Vitamin D levels during the summer. The increase in vitamin D levels during summer is attributed to the sun’s oblique angle of incidence, which is the same reason for latitude [29]. This means that although the sun is closest to Earth in winter among the seasons, the oblique angle at which sunlight enters the atmosphere is high. Additionally, it is predicted that the duration of sunshine hours are related to the seasonality of Vitamin D levels. Indeed, this study confirms the correlation between Vitamin D levels and sunshine hours. The difference in the mean 25(OH)D3 level between summer and winter in this study was 4.3 ng/ml. In the Tokushima, Japan study [19], autumn exhibited the highest 25(OH)D levels in pregnant women, while winter showed the lowest, with a difference of 5.6 ng/ml between the seasons. The disparity in Vitamin D levels between winter and the season with the highest Vitamin D levels in pregnant women was greater in Tokushima than in Hokkaido. Initially, it was predicted that the seasonal variation in vitamin D levels in Hokkaido would be greater due to reduced outdoor activity caused by heavy snowfall, compared to other regions. The average monthly snowfall in Hokkaido during the winter Vitamin D measurement period was 137 cm, compared to 0.8 cm in Tokushima. The snow depth exceeded 10 cm daily throughout the winter [25]. It is sufficiently high to pose a risk to pregnant women. The variation between the highest and lowest seasonal vitamin D levels was smaller in Hokkaido than in Tokushima. However, the low winter vitamin D levels in Hokkaido suggest that snow has a small effect. This disparity between Hokkaido and Tokushima could be attributed to the significantly high autumn vitamin D levels (21.6 ng/mL) in Tokushima. Further research, including behavioral surveys, is necessary to clarify the impact of snow on Vitamin D levels in pregnant women in detail.

A 25(OH)D3 level of 30 ng/ml or higher is considered normal, levels between 20 ng/ml and less than 30 ng/ml indicate a lack of Vitamin D, and levels below 20 ng/ml signify a Vitamin D deficiency [23]. In addition, Vitamin D deficiency can be classified as mild, moderate, or severe [24]. In this study, we employed this classification. From the results of measuring 25(OH)D3, 92.7% of participants had vitamin D deficiency, and four of them had severe deficiency. Considering the previously described effects of low Vitamin D levels on themselves and the fetus, it can be reported that almost all pregnant women in Hokkaido are at high risk. This situation is considered critical. In the survey conducted in Holbæk, Denmark (55.7 degrees north latitude) [17], which is at a higher latitude than Hokkaido, the mean 25(OH)D level in pregnant women was 21.8 nm/ml, approximately 10 nm/ml higher than that in Hokkaido. In the survey conducted in Pennsylvania, USA [28], which is at a similar latitude to Hokkaido, the average 25(OH)D level was 28.1 nm/ml. This level did not meet the normal value but was quite higher than that in Hokkaido, although pregnant women were not included in the comparison. These countries offer products fortified with Vitamin D. In Denmark, certain margarines and spreads are fortified with Vitamin D, while in the USA, it is common for milk to be fortified with this nutrient. In contrast, Japan does not fortify any foods with Vitamin D. In addition, Vitamin D supplements are not widespread in Japan. In this study, only 2.5% of participants used them. While in Pennsylvania, 12.2% of participants used them [28]. Differences in the use of Vitamin D fortification products and supplements were considered a key reason for the varying Vitamin D concentrations between Hokkaido and other cities. Many reports exist on the effects of Vitamin D-fortified products and supplements on Vitamin D concentration. Promoting the use of Vitamin D supplements and introducing Vitamin D-fortified products is essential to safeguard the health of mothers and fetuses in Hokkaido.

This report is the first to examine the Vitamin D levels of pregnant women in Hokkaido, as far as we are aware. It can be said that this is the first general study of Vitamin D levels in pregnant women living in the highest latitude region of Japan. Additionally, the children of the participants in this study can be followed to assess the impact of maternal Vitamin D levels on growth over decades, as JECS is a prospective cohort study. However, in this study, Vitamin D levels were measured at a single time point. The half-life of Vitamin D in the blood is known to be 2–3 weeks. It is hypothesized that Vitamin D levels may vary depending on the timing of measurement, even within the same pregnancy. This issue is currently being reviewed.

## Conclusion

We assessed the levels of 25(OH)D2 and 25(OH)D3 in the serum of 206 pregnant women and analyzed their vitamin D status in Hokkaido, Japan. Four participants had 25(OH)D2 levels above 1 ng/ml, with the highest level being 1.6 ng/ml. The average 25(OH)D3 level was 12.1 ng/ml, the median was 11.0 ng/ml, 92.7% of participants had vitamin D deficiency, and 4 had severe deficiency. Additionally, higher Vitamin D levels were observed in pregnant women during summer and autumn compared to spring and winter, confirming the correlation between Vitamin D levels and sunshine hours. From the study results, the relationship between Hokkaido’s climate and pregnant women’s Vitamin D levels remains unclear, despite nearly all pregnant women in Hokkaido being Vitamin D deficient. To address this critical issue, it is essential to educate pregnant women about the prevalence of vitamin D deficiency and to encourage them to take vitamin D supplements, particularly during the winter and spring months when there are fewer daylight hours and reduced chances to spend time outdoors.

The conclusions of this article are solely the responsibility of the authors and do not represent the official views of the aforementioned government.

## References

[1] Buttriss J. Lanham-New S. Is a vitamin D fortification strategy needed? Nutrition Bulletin, 2020;45:115–122. doi: 10.1111/nbu.12430 32536809 PMC7276911

[2] Holick MF. Vitamin D Deficiency. N Engl J Med. 2007;357(3):266–81. doi: 10.1056/NEJMra070553 17634462

[3] Aranow C. Vitamin D and the Immune System. J Investig Med. 2011;59(6):881. doi: 10.2310/JIM.0b013e31821b8755 21527855 PMC3166406

[4] Teymoori-Rad M, Shokri F, Salimi V, Marashi SM. The interplay between vitamin D and viral infections. Rev Med Virol. 2019;29(2):e2032. doi: 10.1002/rmv.2032 30614127

[5] Ebadi M, Montano-Loza AJ. Perspective: improving vitamin D status in the management of COVID-19. Eur J Clin Nutr. 2020;74(6):856–9. doi: 10.1038/s41430-020-0661-0 32398871 PMC7216123

[6] Holick MF. Medical progress: Vitamin D deficiency. New England Journal of Medicine. 2007;357(3):266–81. 17634462 10.1056/NEJMra070553

[7] Charoenngam N, Holick MF. Immunologic Effects of Vitamin D on Human Health and Disease. Nutrients. 2020;12(7):2097. doi: 10.3390/nu12072097 32679784 PMC7400911

[8] Baggerly CA, Cuomo RE, French CB, Garland CF, Gorham ED, Grant WB, et al. Sunlight and Vitamin D: Necessary for Public Health. J Am Coll Nutr. 2015;34(4):359. doi: 10.1080/07315724.2015.1039866 26098394 PMC4536937

[9] Hayashi F, Takimoto H, Yoshita K, Yoshiike N. Perceived body size and desire for thinness of young Japanese women: a population-based survey. Br J Nutr. 2006;96(6):1154–62. doi: 10.1017/bjn20061921 17181892

[10] Musaei S. The Effect of Pregnancy on the Skin. Eurasian Journal of Chemical, Medicinal and Petroleum Research. 2022;2(1):17–23.

[11] Roth DE, Leung M, Mesfin E, Qamar H, Watterworth J, Papp E. Vitamin D supplementation during pregnancy: state of the evidence from a systematic review of randomised trials. BMJ. 2017;359:j5237. doi: 10.1136/bmj.j5237 29187358 PMC5706533

[12] Harvey NC, Holroyd C, Ntani G, Javaid K, Cooper P, Moon R, et al. Vitamin D supplementation in pregnancy: a systematic review. Health Technol Assess. 2014;18(45):1–189. doi: 10.3310/hta18450 25025896 PMC4124722

[13] De-Regil LM, Palacios C, Lombardo LK, Peña-Rosas JP. Vitamin D supplementation for women during pregnancy. Cochrane Database Syst Rev. 2016;(1):CD008873. doi: 10.1002/14651858.CD008873.pub3 26765344

[14] Litonjua AA, Carey VJ, Laranjo N, Harshfield BJ, McElrath TF, O’Connor GT, et al. Effect of Prenatal Supplementation With Vitamin D on Asthma or Recurrent Wheezing in Offspring by Age 3 Years: The VDAART Randomized Clinical Trial. JAMA. 2016 J;315(4):362–70. doi: 10.1001/jama.2015.18589 26813209 PMC7479967

[15] Yorifuji J, Yorifuji T, Tachibana K, Nagai S, Kawai M, Momoi T, et al. Craniotabes in Normal Newborns: The Earliest Sign of Subclinical Vitamin D Deficiency. J Clin Endocrinol Metab. 2008;93(5):1784–8. doi: 10.1210/jc.2007-2254 18270256

[16] Wilson RL, Leviton AJ, Leemaqz SY, Anderson PH, Grieger JA, Grzeskowiak LE, et al. Vitamin D levels in an Australian and New Zealand cohort and the association with pregnancy outcome. BMC Pregnancy Childbirth. 2018;18(1):1–10. 29925344 10.1186/s12884-018-1887-x PMC6011374

[17] Malm G, Lindh CH, Hansson SR, Källén K, Malm J, Rylander L. Maternal serum vitamin D level in early pregnancy and risk for preeclampsia: A case-control study in Southern Sweden. PLoS One. 2023;18(2):e0281234. doi: 10.1371/journal.pone.0281234 36749741 PMC9904465

[18] Orvik AB, Andersen MR, Bratholm PS, Hedengran KK, Ritz C, Stender S, et al. Variation in plasma 25-hydroxyvitamin D2 and D3 in normal pregnancy with gestational age, sampling season, and complications: A longitudinal cohort study. PLoS One. 2020;15(4):e0231657. doi: 10.1371/journal.pone.0231657 32302333 PMC7164833

[19] Sogawa E, Kaji T, Nakayama S, Yoshida A, Yonetani N, Maeda K, et al. Seasonal variation of serum 25(OH) vitamin D levels in maternal and umbilical cord blood in Japanese women. The Journal of Medical Investigation. 2019;66(1.2):128–33. doi: 10.2152/jmi.66.128 31064925

[20] Kanatani KT, Nakayama T, Adachi Y, Hamazaki K, Onishi K, Konishi Y, et al. High frequency of vitamin D deficiency in current pregnant Japanese women associated with UV avoidance and hypo-vitamin D diet. PLoS One. 2019;14(3):e0213264. doi: 10.1371/journal.pone.0213264 30830935 PMC6398852

[21] Takatani T, Kunii Y, Satoh M, Eguchi A, Yamamoto M, Sakurai K, et al. Vitamin D Metabolite Ratio in Pregnant Women with Low Blood Vitamin D Concentrations Is Associated with Neonatal Anthropometric Data. Nutrients. 2022;14(11):2201. doi: 10.3390/nu14112201 35684001 PMC9182679

[22] Nakajima H, Sakamoto Y, Honda Y, Sasaki T, Igeta Y, Ogishima D, et al. Estimation of the vitamin D (VD) status of pregnant Japanese women based on food intake and VD synthesis by solar UV-B radiation using a questionnaire and UV-B observations. J Steroid Biochem Mol Biol. 2023;229:106272. doi: 10.1016/j.jsbmb.2023.106272 36775044

[23] Holick MF, Binkley NC, Bischoff-Ferrari HA, Gordon CM, Hanley DA, Heaney RP, et al. Evaluation, Treatment, and Prevention of Vitamin D Deficiency: an Endocrine Society Clinical Practice Guideline. J Clin Endocrinol Metab. 2011;96:1911–1930. doi: 10.1210/jc.2011-0385 21646368

[24] Gani LU, How CH. Vitamin D deficiency. Singapore Med J. 2015;56(8):433. 26311908 10.11622/smedj.2015119 PMC4545131

[25] Japan Meteorological Agency. Historical weather date search. 2023 [cited 21 March 2023]. In: Japan Meteorological Agency [internet]. Tokyo: Japan Meteorological Agency. Available from: https://www.data.jma.go.jp/obd/stats/etrn/index. Accessed 21 Mar 2023.

[26] Machida H, Tsunekawa K, Sakamaki K, Kimura T, Abe Y, Murakami M. Sex Differences in Serum 25-hydroxyvitamin D Reflect Differences in 25-hydroxyvitamin D3 Levels but not in D2 Levels. The Kitakanto Medical Journal. 2023;73:17–21.

[27] Rahat B, Hamid A, Bagga R, Kaur J. Folic Acid Levels During Pregnancy Regulate Trophoblast Invasive Behavior and the Possible Development of Preeclampsia. Front Nutr. 2022;9 847136. doi: 10.3389/fnut.2022.847136 35578613 PMC9106796

[28] Leary PF, Zamfirova I, Au J, McCracken WH. Effect of latitude on vitamin D levels. Journal of the American Osteopathic Association. 2017;117(7):433–9. doi: 10.7556/jaoa.2017.089 28662556

[29] Holick MF. Sunlight and vitamin D for bone health and prevention of autoimmune diseases, cancers, and cardiovascular disease. Am J Clin Nutr. 2004;80(6):1678S–1688S. doi: 10.1093/ajcn/80.6.1678S 15585788

[30] van der Mei IAF, Ponsonby AL, Engelsen O, Pasco JA, McGrath JJ, Eyles DW, et al. The high prevalence of vitamin D insufficiency across Australian populations is only partly explained by season and latitude. Environ Health Perspect. 2007;115(8):1132–9. doi: 10.1289/ehp.9937 17687438 PMC1940076

[31] Chersich MF, Pham MD, Areal A, Haghighi MM, Manyuchi A, Swift CP, et al. Associations between high temperatures in pregnancy and risk of preterm birth, low birth weight, and stillbirths: systematic review and meta-analysis. BMJ. 2020;371:m3811. doi: 10.1136/bmj.m3811 33148618 PMC7610201

